# Synthesis, Structural and Optical Properties of ZrBi_2_Se_6_ Nanoflowers: A Next-Generation Semiconductor Alloy Material for Optoelectronic Applications

**DOI:** 10.1021/acsomega.2c02666

**Published:** 2022-09-01

**Authors:** Rahul Aher, Ashvini Punde, Pratibha Shinde, Shruti Shah, Vidya Doiphode, Ashish Waghmare, Yogesh Hase, Bharat R. Bade, Yogesh Jadhav, Mohit Prasad, Habib M. Pathan, Shashikant P. Patole, Sandesh R. Jadkar

**Affiliations:** † Department of Physics, Savitribai Phule Pune University, Pune 411007, India; ‡ Symbiosis Center for Nanoscience and Nanotechnology, Symbiosis International Deemed University, Pune 412115, India; § Department of Applied Science and Humanities, Pimpri Chinchwad College of Engineering, Nigdi, Pune 411004, India; ∥ Department of Physics, Khalifa University of Science and Technology, Abu Dhabi 127788, UAE

## Abstract

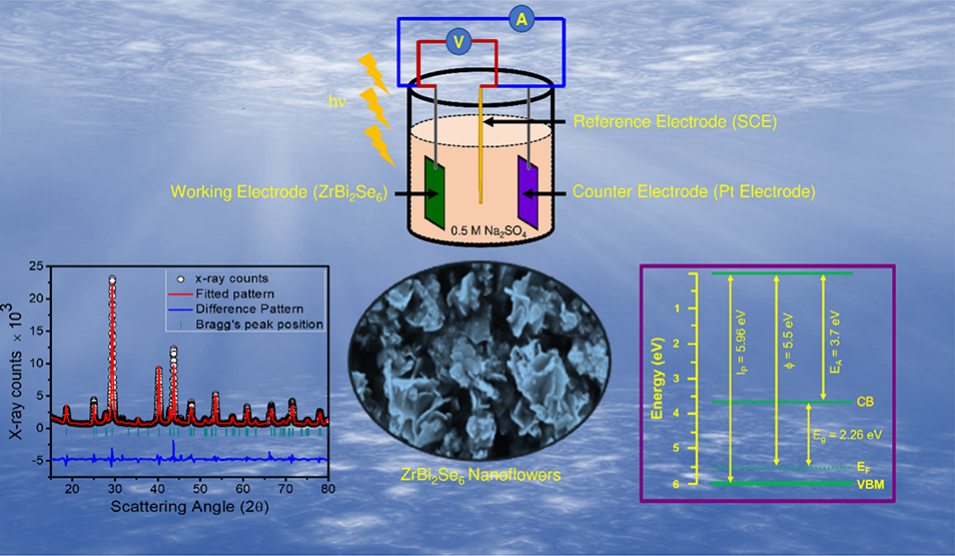

ZrBi_2_Se_6_ nanoflower-like morphology was successfully prepared using a solvothermal method, followed by a quenching process for photoelectrochemical water splitting applications. The formation of ZrBi_2_Se_6_ was confirmed by field emission scanning electron microscopy (FE-SEM), X-ray diffraction (XRD), Raman spectroscopy, and X-ray photoelectron spectroscopy (XPS). The estimated value of work function and band gap were found to be 5.5 and 2.26 eV measured using diffuse reflection spectroscopy and ultraviolet photoelectron spectroscopy, suggesting the potential candidate for water splitting. The highest current density of 9.7 μA/cm^2^ has been observed for the ZrBi_2_Se_6_ photoanode for the applied potential of 0.5 V vs SCE. The flat-band potential value was −0.46 V, and the 1.85 nm width of the depletion region is estimated from the Mott–Schottky (MS) analysis. It also reveals that the charge carrier density for the ZrBi_2_Se_6_ nanoflowers is 4.8 × 10^15^ cm^–3^. The negative slope of the MS plot indicates that ZrBi_2_Se_6_ is a p-type semiconductor. It was observed that ZrBi_2_Se_6_ nanoflowers had a high charge transfer resistance of ∼730 kΩ and equivalent capacitance of ∼40 nF calculated using electrochemical impedance spectroscopy (EIS) measurements. Using chronoamperometry, the estimated rise time and decay time were 50 ms and 0.25 s, respectively, which reveals the fast photocurrent response and excellent PEC performance of the ZrBi_2_Se_6_ photoanode. Furthermore, an attempt has been made to explain the PEC activity of ZrBi_2_Se_6_ nanoflowers using an energy band diagram. Thus, the initial results on ZrBi_2_Se_6_ nanoflowers appear promising for the PEC activity toward water splitting.

## 1 Introduction

The production of hydrogen is a renewable, clean, and carbon-free process. It shows a remarkable energy density of (140 MJ/kg),^1^ which will solve the current energy crisis and environmental deterioration due to fast industrialization and population enlargement. Hydrogen is an environmentally friendly source of energy. It has been applied widely in fuel-cell electric vehicles refining petroleum, producing fertilizers, treating metals, and processing foods.^2−4^ On the other hand, traditional fossil fuels release toxic and greenhouse gases and will soon exhaust in the upcoming few decades. Thus, it is high time to search for a way to generate energy with minimum damage to mother nature, so hydrogen is one of the most promising candidates. Many efforts have been made to produce hydrogen as fuel throughout the world. Cheng *et al.*(5) obtained solar to hydrogen (STH) efficiencies of 18.5 and 19.3% in neutral and acidic electrolytes, respectively. Karuturi *et al*.^6^ have developed self-driven solar water splitting with 17.6% STH efficiency. Varadhan *et al.*(7) fabricated a monolithically integrated photoelectrochemical (PEC) cell. They achieved a remarkable STH efficiency of ∼9% and better stability of ∼150 h with the photoanode through epitaxial lift-off and transferring of the grown InGaP/GaAs into a robust Ni substrate.

Although steam methane reforming at a low temperature produces extensive hydrogen, it liberates a large amount of greenhouse gas.^8^ Many green technologies such as electrocatalytic,^9,10^ photocatalytic,^11−13^ and the thermo-photo hybrid catalytic water splitting^14−16^ have been attempted in the last decade for efficient hydrogen production from water. The PEC water splitting is highly promising and utilizes abundant solar and water resources for hydrogen production.^17,18^ The search for photoelectrode material and PEC for hydrogen production became a research hotspot after the discovery of TiO_2_ as a photoelectrode to produce hydrogen using UV irradiation treatment by Fujishima and Honda in 1972.^19^ Therefore, the growth and exploration of active and effective semiconductor materials as photoelectrodes/photocatalysts for the production of H_2_ are sought. However, one of the most challenging parts is identifying suitable semiconductor electrodes with high conversion efficiency, good electrical conductivity, and optical conductivity with a low electron–hole recombination rate to realize smooth charge mobility. Many bulk semiconductor nanomaterials show limited photoanode application. Therefore, they strongly need further modifications or search for new semiconductor nanomaterials, alloys/composites with the appropriate optoelectronic properties, optimum band gap, high absorption coefficient, band edge positions, etc.

Even with suitable properties, devices show poor performance because the main issue of most semiconductors is the recombination rate of photogenerated electron–hole pairs, which dominates the electron transfer rate. The new class of materials, such as topological insulators and 2D layered nanomaterials, has drawn significant interest because of the high specific surface area, fast electron transfer rate, and good light harvesting property of metallic surfaces.^20^ The ultrathin geometrical structure of such layered materials shows many exciting features in the electronic and optical properties domain due to their mixed ionic-covalent characteristics.^21^ The hybrid ionic-covalent characteristics are favorable for efficient charge carrier transport. These fundamental characteristics help to enhance electronic and dielectric properties because such states with mixed ionic-covalent characters have dispersive conduction and valence bands with a large static dielectric constant.^22^ Additionally, it is advantageous because it reduces carrier scattering and trapping because of the strong screening of impurities and charged defects.^21,23,24^ These materials show excellent surface properties and satisfy two essential PEC properties for water splitting to generate hydrogen. The first is the ideal optical band gap (∼2.0–3.00 eV). The second is that the valence band maximum energy must be more positive than that of the oxidation potential of O_2_/H_2_O (1.23 V vs NHE), and the conduction band minimum energy must be more negative than that of the reduction potential of H^+^/H_2_ (0 V vs NHE). Zirconium triselenide (ZrSe_3_) and bismuth selenide (Bi_2_Se_3_) are layered semiconductors and are predicted to be topological insulators.^25−28^ Their fundamental band gap is indirect. However, the direct gap presents strong excitonic effects and a mixed covalent and ionic character inside the layer, resulting in excellent conduction properties through the layered structures.^29,30^ Therefore, such semiconductors have been extensively studied for device fabrication. Wu and his group^31^ proposed the use of ZrSe_3_ nanoflakes for optical limiters. Based on the results obtained by Xiong *et al.*,^29^ ZrSe_3_ nanobelts were used in photodetectors under a range of visible light conditions. Likewise, Bi_2_Se_3_ thin films showed good rectifying properties suitable for photodetectors in the UV–visible–NIR region.^32^ Desai *et al.*(33) employed it in solar cells and obtained the highest power conversion efficiency of 0.14%. Recently, nanoflowers of Bi_2_Se_3_ have been used with TiO_2_ as photoanodes for photoelectrochemical water splitting.^33^

There is a great interest in investigating the ZrSe_3_-Bi_2_Se_3_ system by exploring the Zr–Bi–Se mixed alloy system, which provides an opportunity for a tailorable and highly customizable structure with expanded material choices and structural property variations. However, there is no report in the literature on such a Zr–Bi–Se mixed alloy system to date. With this motivation, an attempt has been made to synthesize a novel semiconductor alloy, zirconium bismuth selenide (ZrBi_2_Se_6_), using the solvothermal method. The formation of ZrBi_2_Se_6_ was confirmed by XRD, Raman spectroscopy, and XPS analysis. In addition, FE-SEM has established the nanoflower morphology of ZrBi_2_Se_6_. Finally, the PEC properties of ZrBi_2_Se_6_ photoanodes were explored for water splitting under the illumination of white light. We have found that ZrBi_2_Se_6_ can be a good candidate for PEC water splitting over traditional photoanodes.

## 2 Experimental Section

### 2.1 Chemicals and Materials

Zirconium oxychloride octahydrate [ZrOCl_2_.8H_2_O] and bismuth acetate [(CH_3_CO_2_)_3_Bi], oleic acid, 1-octadecane, selenourea, and ethanol were used directly as purchased from Sigma Aldrich.

### 2.2 Synthesis of ZrBi_2_Se_6_ Nanoflowers and Thin Films

The two-step strategy has been used to synthesize zirconium bismuth selenide (ZrBi_2_Se_6_) nanoflowers. In the typical synthesis procedure, zirconium oxychloride octahydrate and bismuth acetate are added to 16 mL of oleic acid. The molar ratio of Zr:Bi was maintained at about 1:2. Zr and Bi salts were dissolved at 100 °C in the presence of Argon gas in a round bottom flask of 100 mL. To make the solution homogeneous, the mixture was heated for 1 h with continuous stirring at 800 rpm. In this homogeneous solution, 0.73 mmol selenourea and 25 mL of 1-octadecene were added as selenium sources, and then finally, the solution was transferred to a 100 mL Teflon-lined stainless steel autoclave. The autoclave was maintained at 180 °C for 24 h, followed by quenching in an ice bath. The resultant residue was washed with absolute ethanol several times. The black-brownish precipitate dried in the tube furnace at 200 °C for 12 h under argon gas. After drying, black powder of ZrBi_2_Se_6_ was obtained and used for further characterization. The thin films of ZrBi_2_Se_6_ have been prepared by simple electrophoretic deposition using two-electrode systems (Pt and FTO) at 25 mV potential for a 10 min duration.

### 2.3 Material Characterization

In the present work, various complementary techniques and spectroscopies have been carried out for structural, optical, and band structure analyses of as-synthesized ZrBi_2_Se_6_ nanoflowers. The XRD pattern of the prepared ZrBi_2_Se_6_ nanoflowers was performed using a Bruker D8 Advance X-ray diffractometer (Germany, CuKα radiation of 1.54056 Å). A JASCO, V-670 UV–visible near-infrared (UV–Vis–NIR, Japan) spectrophotometer was recorded for the diffuse reflection spectra (DRS) in the 200–800 nm range. The optical absorption and band gap of ZrBi_2_Se_6_ were estimated from the DRS spectra. The surface morphology of ZrBi_2_Se_6_ nanoflowers was studied using a scanning electron microscope (FEI Nova NanoSem 450 FE-SEM). XPS analysis of ZrBi_2_Se_6_ nanoflowers with Al Kα (1486.6 eV) radiation was recorded using Thermo Scientific, Kα, UK machine with a resolution of 0.1 eV. The binding energy was corrected for specimen charging by referencing C 1s to 284.6 eV. The ultraviolet photoemission spectroscopy (UPS) spectra were recorded using a comprehensive facility for AIPES measurements at the INDUS-2 synchrotron source at Raja Ramanna Centre for Advanced Technology (RRCAT), Indore (India). The *I*–*V* characteristics of the deposited film were recorded using a potentiostat (Metrohm Autolab-PGSTAT302N) and a solar simulator (PEC-L01) of 100 mW/cm^2^ intensity with AM 1.5. Electrochemical impedance spectroscopy has been studied under illumination using the same potentiostat, between 0.1 and 100 kHz. The Mott–Schottky curves were performed at the frequency of 100 Hz under dark conditions. To study the PEC activity of ZrBi_2_Se_6_ nanoflower photoanodes, linear sweep voltammetry (LSV) was used. The three-electrode system was used for the measurements at the potential region of −1.0 to 1.0 V vs SCE in 0.5 M Na_2_SO_4_ (pH = 7) electrolyte under illumination and dark conditions.

## 3 Results and Discussion

### 3.1 X-ray Diffraction Analysis

**Figure 1:**
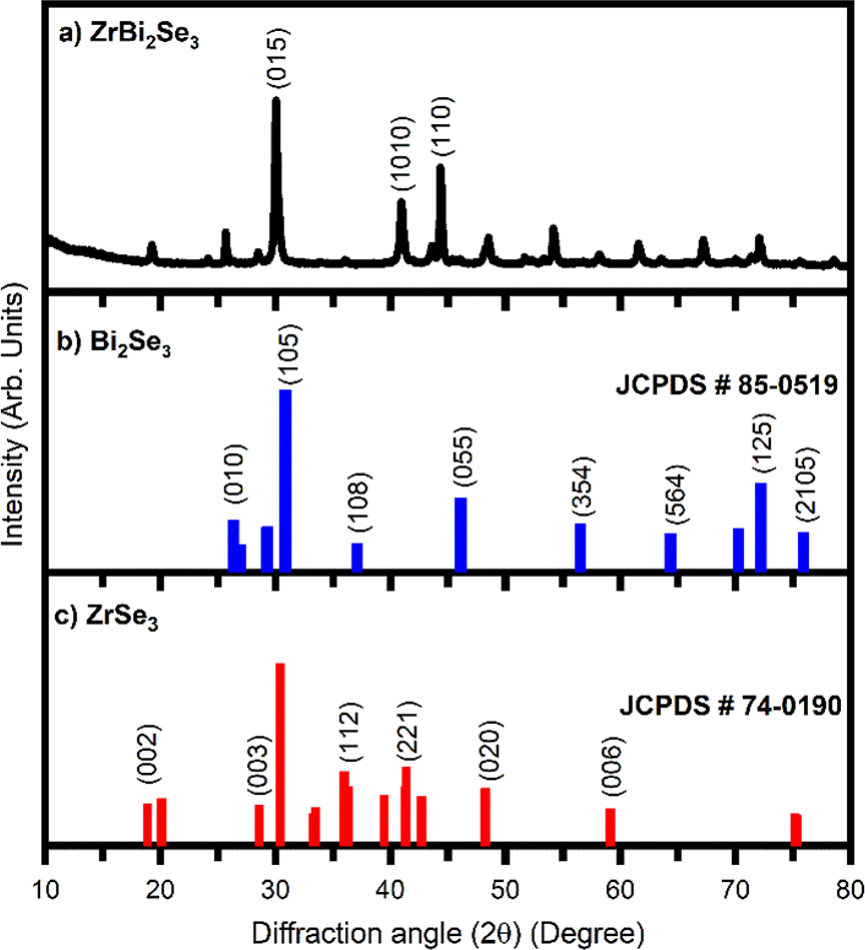
XRD pattern of (a) ZrBi_2_Se_6_ powder obtained using the solvothermal method, (b) JCPDS XRD pattern of Bi_2_Se_3_, and (c) JCPDS XRD pattern of ZrSe_3_.

In the X-ray diffraction (XRD) pattern, the peak position and intensity provide the crystal structure and unit cell information. Furthermore, the atom’s position can also be predicted from the intensities of the peaks. Figure 1a depicts the XRD analysis of ZrBi_2_Se_6_ powder obtained using the solvothermal method. For comparison, the JCPDS XRD patterns of Bi_2_Se_3_ [Figure 1b] and ZrSe_3_ [Figure 1c] are also included in Figure 1. The recorded XRD pattern of ZrBi_2_Se_6_ reveals the contribution of major peaks from Bi_2_Se_3_ and Zr_2_Se_3_. It has been reported that if the electronegativity difference between the two bonded atoms is 0.5–2.1, then the bonds formed between them are polar covalent. The electronegativity difference (Δ*E*_N_) values between Bi–Se, Zr–Se, and Zr–Bi are 0.53, 1.22, and 0.69, respectively, and the ionic radii of the Bi, Zr, and Se atoms are 230, 230, and 190 nm, respectively. It results in the formation of polar covalent bonds between Bi–Se, Zr–Se, and Zr–Bi. Bi_2_Se_3_ and ZrSe_3_ have a hexagonal crystal structure, and both materials belong to layered structured families. Therefore, the atomic layer polar covalent interaction in the plane and van der Waals interaction out of the plane promotes the epitaxial growth of Zr_2_Se_3_ on Bi_2_Se_3_. Furthermore, due to Bi’s dominant valency and electronegativity, the formation of the Bi_2_Se_3_-like structure is more prominent than any other possible structure. These results show the formation of ZrBi_2_Se_6_ by the solvothermal method.

**Figure 2:**
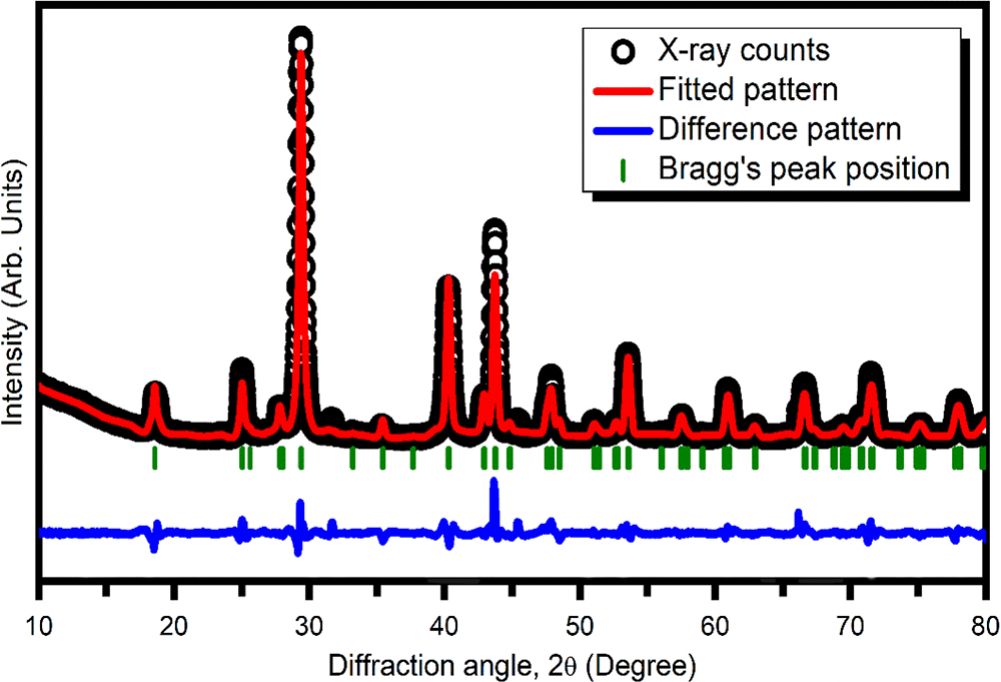
Rietveld refinement of ZrBi_2_Se_6_ powder obtained using the solvothermal method.

The average crystallite size and lattice strain of ZrBi_2_Se_6_ were measured using the Rietveld refinement method. Figure 2 shows the Rietveld refinement for the ZrBi_2_Se_6_ powder. For Rietveld refinement, various parameters, viz., lattice parameters, background points, zero-point parameter, scale parameter, overall thermal parameter, and half-width parameters, were varied during refinement. The analysis confirms the formation of hexagonal ZrBi_2_Se_6_ with the *R*3*m* space group. The interplanar distance for hexagonal ZrBi_2_Se_6_ is given by
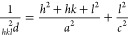
1

The estimated values of the lattice constants for the ZrBi_2_Se_6_ hexagonal structure are *a* = *b* = 4.14 Å, *c* = 28.66 Å and *V* = 491.2 (Å)^3^. The Bi_2_Se_3_ single crystals belong to the family of hexagonal systems with lattice parameters *a* = 4.14 Å, *c* = 28.62 Å, and *V* = 424.6 (Å)^3^,^34^ whereas ZrSe_3_ has the monoclinic structure with lattice parameters of *a* = 5·13 Å, *b* = 3·61 Å, and *c* = 9·01 Å and *V* = 193·5 (Å)^3^.^35^ In the comparison, the lattice of the ZeBi_2_Se_6_ has been expanded.

The values of full width at half-maximum (FWHM) with Bragg’s angle derived from Rietveld refinement were used for Williamson–Hall (W-H) analysis to estimate lattice strain (ε) and crystalline size (D) using^36^
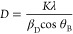
2
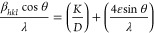
3where λ is the wavelength in Å, *K* is the shape factor (0.89), θ_B_ is the Bragg angle, and FWHM is represented by β_*hkl*_ in radians. The average crystallite size and lattice strain are estimated by plotting β_*hkl*_cosθ vs 4 sinθ.

**Figure 3:**
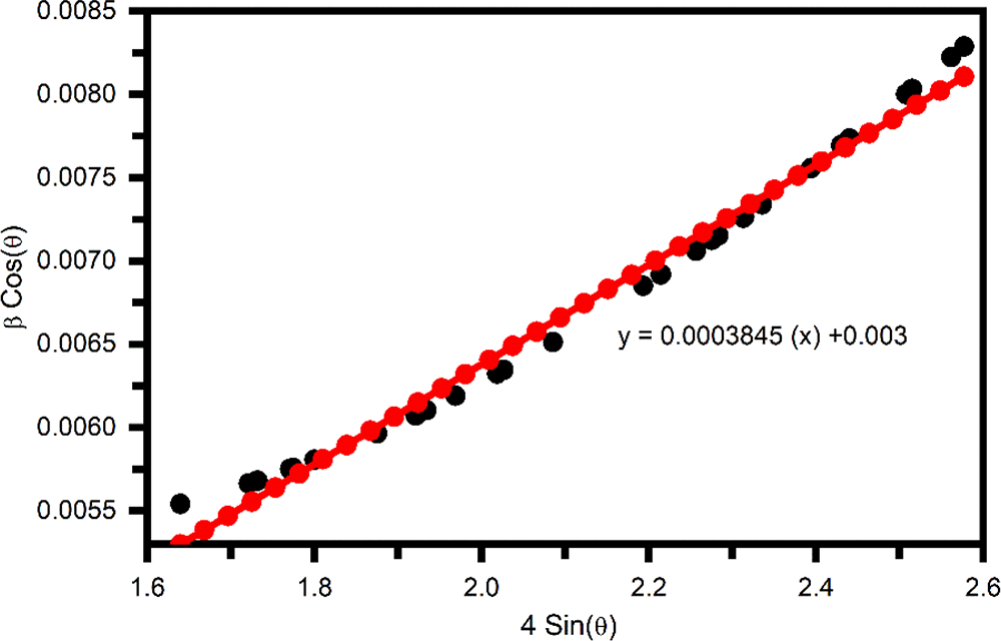
Williamson–Hall (W-H) plots for ZrBi_2_Se_6_ powder obtained using the solvothermal method.

Figure 3 shows the Williamson–Hall plot for ZrBi_2_Se_6_, which reveals the case of pure strain broadening for the present ZrBi_2_Se_6_ nanoflowers. The lattice strain and average crystallite size calculated from the slope and the fitted line intercepts are 0.0030 and 35.97 nm, respectively.

### 3.2 Raman Spectroscopy

**Figure 4:**
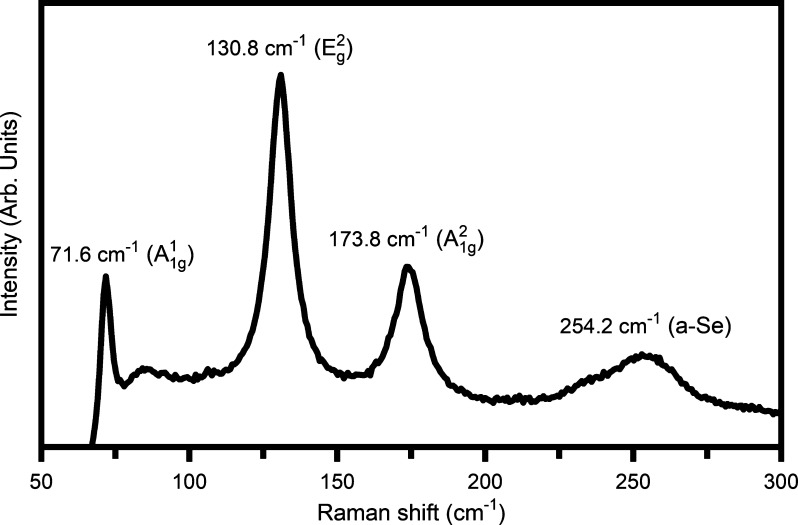
Raman spectra of the ZrBi_2_Se_6_ nanoflowers synthesized using the solvothermal method.

Raman spectroscopy is an effective technique to detect the composition, polytypism, number of layers, strain, and material defects.^37^ Furthermore, it provides vital information such as the symmetry–asymmetry nature of the bond vibrations, molecular orientations, isotropic and ordered phases, and crystalline and amorphous phases.^38,39^ Furthermore, the formation of ZrBi_2_Se_6_ was confirmed through Raman spectroscopy. Figure 4 shows the Raman spectra for ZrBi_2_Se_6_ synthesized using the solvothermal method.

The unpolarized Raman spectrum shows peaks at ∼71.6, 130.8, 170.8, and 254.2 cm^–1^ indexed to the A_1g_^1^, E_g_^2^, and A_1g_^2^ optical phonon modes of α-Bi_2_Se_3_ in the as-synthesized ZrBi_2_Se_6_ powder. These results are consistent with the previously reported Raman spectra for α-Bi_2_Se_3._^40^ The peak ∼254.2 cm^–1^ is associated with a-Se, indicating that the synthesized ZrBi_2_Se_6_ is selenium-rich.^41,42^ The non-existence of low-frequency modes (<50 cm^–1^) in the Raman spectra may be due to the high Rayleigh background and instrument limitation. These results follow the previously reported literature for ZrSe_2_^37^ and Bi_2_Se_3._^43^

### 3.3 X-ray Photoelectron Spectroscopy (XPS)

**Figure 5:**
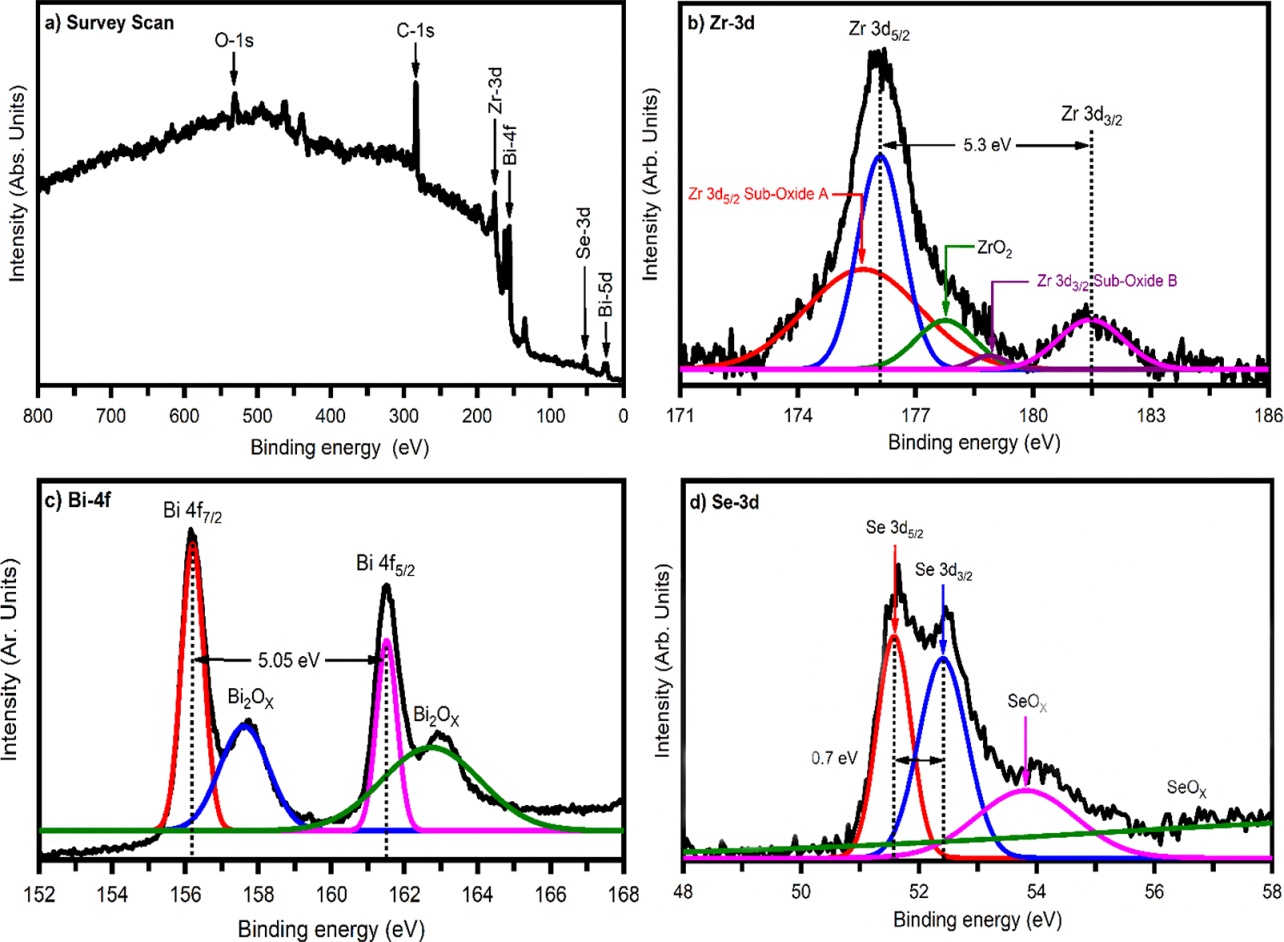
XPS spectra of the ZrBi_2_Se_6_ sample: (a) Survey scan from 0 to 800 eV, (b) narrow scan for Zr 3d in the range 171–186 eV, (c) narrow scan for Bi 4f in the range 152–168 eV, and (d) narrow scan for Se 3d in the range 48–58 eV.

The electronic structure and chemical properties of the ZrBi_2_Se_6_ nanoflowers were analyzed qualitatively using high-resolution X-ray photoelectron spectroscopy (XPS). Figure 5a shows the survey XPS spectra of the ZrBi_2_Se_6_ sample. As seen, the peaks corresponding to zirconium [Zr(3d)], bismuth [Bi(4f)], selenium [Se(3d)], carbon [C(1s)], and oxygen [O(1s)] appear in the XPS spectra.

Figure 5b–d shows the narrow scan XPS spectra for Zr 3d, Bi 4f, and Se 3d elements. In Figure 5b, two peaks were observed for the narrow XPS spectra of Zr 3d. The peak at ∼176.09 eV is Zr 3d_5/2_, and the peak at ∼181.51 eV is due to Zr 3d_3/2_ with spin–orbit splitting separation at 5.32 eV. It indicates the existence of Zr^4+^ in the as-prepared ZrBi_2_Se_6_ sample.^44,45^ Sometimes, another doublet set with Zr^4+^ can coexist at high-energy parts. The broadening in the core-level Zr 3d_5/2_ peak is mainly due to the suboxide A peak found at the lower energy side and the ZrO_2_ and suboxide B peaks found at the higher energy side, which is realized after deconvolution of the spectra. The Bi 4f spectrum contains two peaks at ∼156.20 and ∼161.25 eV accomplice with Bi 4f_7/2_ and Ag 4f_5/2_, respectively [Figure 5c]. These two peaks were disjointed with an energy value of ∼5.05 eV. The splitting in Bi 4f peaks can be attributed to the surface polarization of Se and Zr/Se in ZrBi_2_Se_6_. It confirms that the Bi exists with a +3 oxidation state. Two satellite peaks at ∼157.55 and ∼162.68 eV are found at lower binding energy sides. These satellite peaks are the secondary order XPS peaks with low intensity, close to the intense parent peak. These low-energy satellite peaks can appear due to the increased localized relaxed orbital occupation probability because of the charge transferred from a ligand orbital and an abrupt change in Coulombic potential as the photo rejected electron passes through the valence band.

On the other hand, the observed splitting and suppressed binding energy of the Se 3d peak is due to various chemical environments. The chemical shift in the Se core electron depends on the neighboring atom’s nature and the element’s oxidation state. Electronegativity also plays a vital role in the observed spin–orbit splitting (∼0.7 eV) for 3d_5/2_ and 3d_3/2_. Two suboxide peaks (SeO_*X*_) are observed at the higher energy side near the parent Se 3d_3/2_ peak. These results confirm that the Se is present in their −2 oxidation state in the ZrBi_2_Se_6_ sample. Furthermore, these results show that ZrBi_2_Se_6_ is stable and is not easily oxidized under atmospheric conditions, favoring its practical applications.

### 3.4 Field Emission-Scanning Electron Microscopy (FE-SEM) Analysis

**Figure 6:**
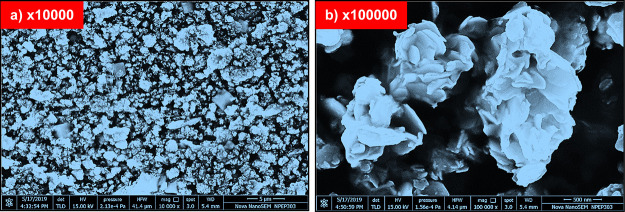
FESEM images of as-synthesized ZrBi_2_Se_6_ powder at different magnifications: (a) ×10,000 and (b) ×100,000.

The surface morphology of solvothermally grown ZrBi_2_Se_6_ powder was determined using FE-SEM. Figure 6a,b shows FE-SEM images of ZrBi_2_Se_6_ powder at ×10000 and ×100000 resolutions. It was observed that the formation is well-organized and with flower-like nanostructures with diameters of ∼0.6–0.8 μm.

Furthermore, after careful observation of the FE-SEM images, it is observed that ZrBi_2_Se_6_ evolved vertically oriented sharp nano pedals with porous microcavities inside it that are assembled into nanoflowers with increased surface area.

### 3.5 Ultraviolet Photoelectron Spectroscopy (UPS) Analysis

**Figure 7:**
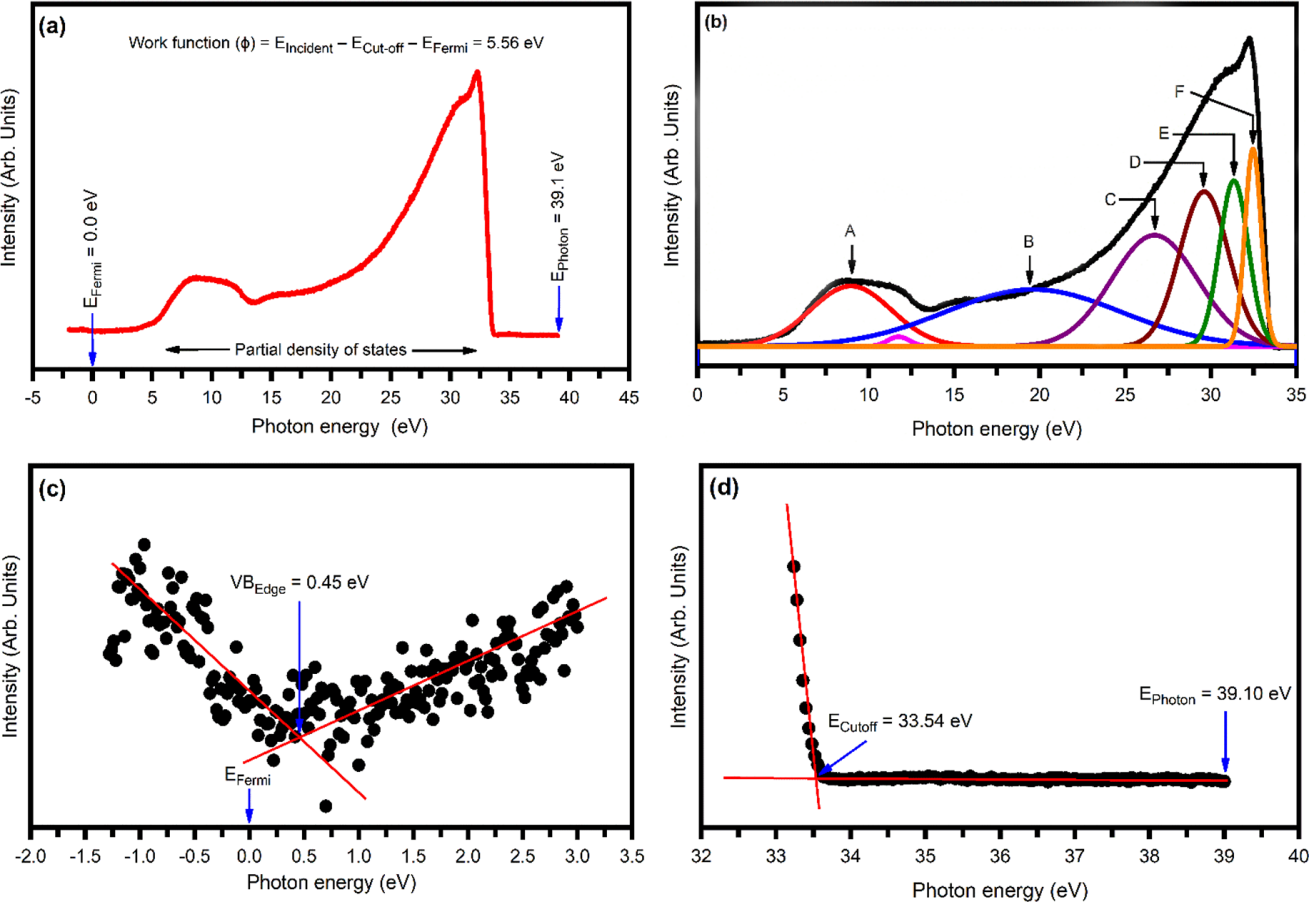
(a) Typical UPS spectra of ZrBi_2_Se_6_ nanoflowers obtained at an incident photon energy of 40 eV. (b) Deconvoluted UPS spectra. (c) Magnified view of the valence band (VB) region to estimate the valence band edge (VB_Edge_). (d) Magnified view of the cutoff region to estimate cut off energy (*E*_Cutoff_).

It is necessary to characterize the ZrBi_2_Se_6_ nanoflower–metal interface to integrate bulk nanomaterial into effective devices. For this purpose, it is essential to know the surface parameters such as work function, ionization potential, electron affinity, Fermi level, etc. Furthermore, band bending mainly influences the interface property caused by a difference in the work functions between the semiconductor and metal. Hence, it plays a crucial role in device operation. Thus, ultraviolet photoelectron spectroscopy (UPS) was used to calculate the work function (ϕ) and predict the valence band structure of ZrBi_2_Se_6_ nanoflowers. The typical UPS spectra of ZrBi_2_Se_6_ nanoflowers measured at an incident photon energy ∼39.10 eV is shown in Figure 7a.

Figure 7b represents the deconvoluted UPS spectra of the ZrBi_2_Se_6_ nanoflowers. Figure 7c,d represents the magnified view of the valence band maxima (VBM) and the cut-off energy regions of the UPS spectra, respectively. The deconvoluted UPS spectra of the ZrBi_2_Se_6_ nanoflowers consist of six Gaussian peaks at ∼9.00, ∼19.41, ∼26.67, ∼29.56, ∼31.32, and ∼32.45 eV. Peak A is mainly attributed to the Se *p*-orbital. Peaks B, C, and D are contributed from the Bi *p*-orbital, Zr *d*-orbital, and Bi *p*-orbital. The last E and F peaks may result from strong hybridized Bi and Se states contributing to the p-orbital.^25,46,47^

The work function can be calculated using the equation^48^
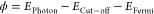
4

where *E*_Photon_ = *h*ν = incident photon energy (39.1 eV), *E*_*F*ermi_ is the Fermi energy (0.0 eV, calibrated by using the Au standard sample), and *E*_Cut-off_ is the cut-off energy (33.6 eV) calculated from the UPS spectra. Thus, the value of the work function of ZrBi_2_Se_6_ nanoflowers was ∼5.56 eV. The estimated work function, ionization potential, and electron affinity of the ZrBi_2_Se_6_ nanoflowers are summarized in Table 1.

**Table 1:** Estimated Work Function (ϕ), Ionization Potential (*I*_P_), and Electron Affinity (χ_e_) of the ZrBi_2_Se_6_ Nanoflowers

material	VBM edge (eV)	*I*_p_ (eV)	χ_e_ (eV)	ϕ (eV)
ZrBi_2_Se_6_	0.45	5.96	3.7	5.5

### 3.6 Diffuse Reflectance Spectroscopy (DRS)

**Figure 8:**
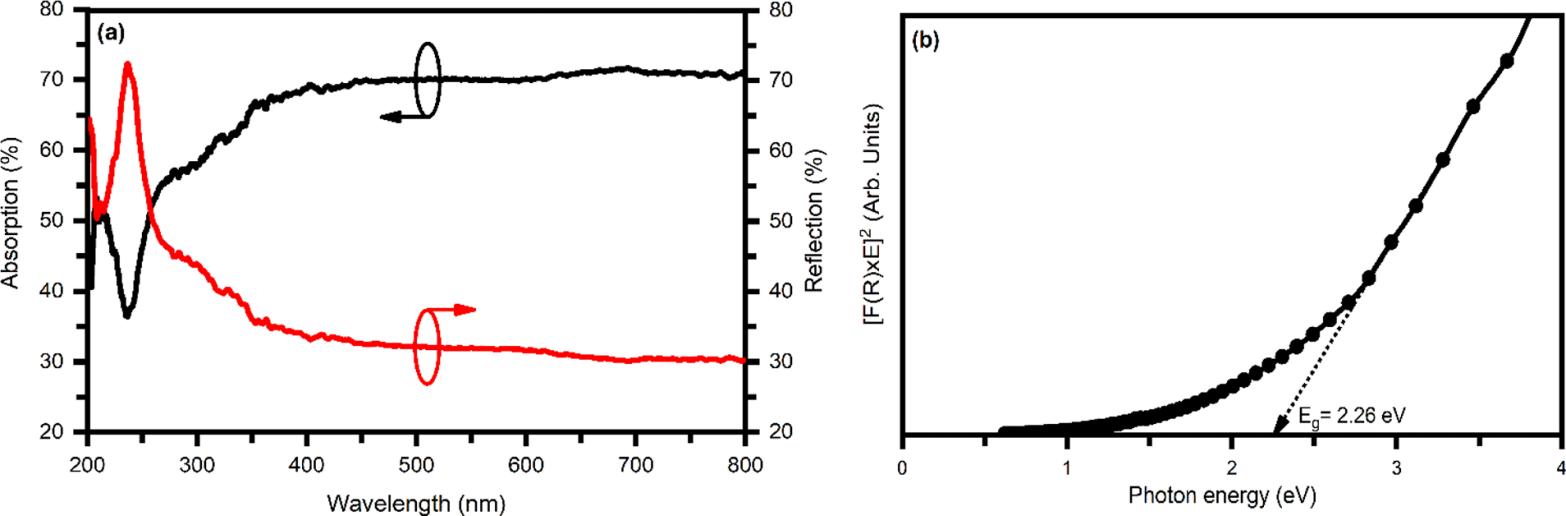
(a) Absorption and reflection plot as a function of wavelength of ZrBi_2_Se_6_ nanoflowers. (b) Tauc plot used to evaluate the band gap of ZrBi_2_Se_6_ using the Kubelka–Munk transformation.

DRS is generally applied to highly light scattering materials and absorbing particles in a matrix. Therefore, the photon energy-dependent optical properties of ZrBi_2_Se_6_ nanoflowers were studied using diffuse reflectance spectroscopy. Figure 8a shows the typical reflectance and absorbance plot of ZrBi_2_Se_6_ nanoflowers as a function of wavelength.

If *t* and *A* are the thickness and absorbance of the film, the absorption coefficient (α) was measured by using Beer–Lambert’s law [^49^],
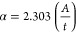
5

It has been observed that the ZrBi_2_Se_6_ nanoflowers have more than a >10^4^ cm^–1^ absorption coefficient in the visible spectra region, indicating the high probability of direct transition.

To calculate the optical band gap of ZrBi_2_Se_6_ nanoflowers, the values of diffuse reflectance *R* have been changed to equivalent emission extinction coefficients, [*F*(*R*)], by using the Kubelka–Munk transformation,^49^
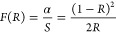
6

Using DRS, the analogous Tauc plots may be derived from the Kubelka–Munk function, *F*(*R*), according to equation,^50^
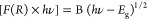
7where *B* is a proportionality constant. The optical band gap is measured by plotting the tangential line to the axis of the photon energy (*E* = *h*υ) in the plot of [*F*(*R*) × *h*υ]^1/2^ as a function of *h*υ (Tauc plot). Figure 8b represents the Tauc plot for the ZrBi_2_Se_6_ sample. The evaluated band gap value for ZrBi_2_Se_6_ is ∼2.26 eV, which lies in the ideal band gap range for catalytic water splitting.^51^

## 4 Photoelectrochemical (PEC) Analysis

**Figure 9:**
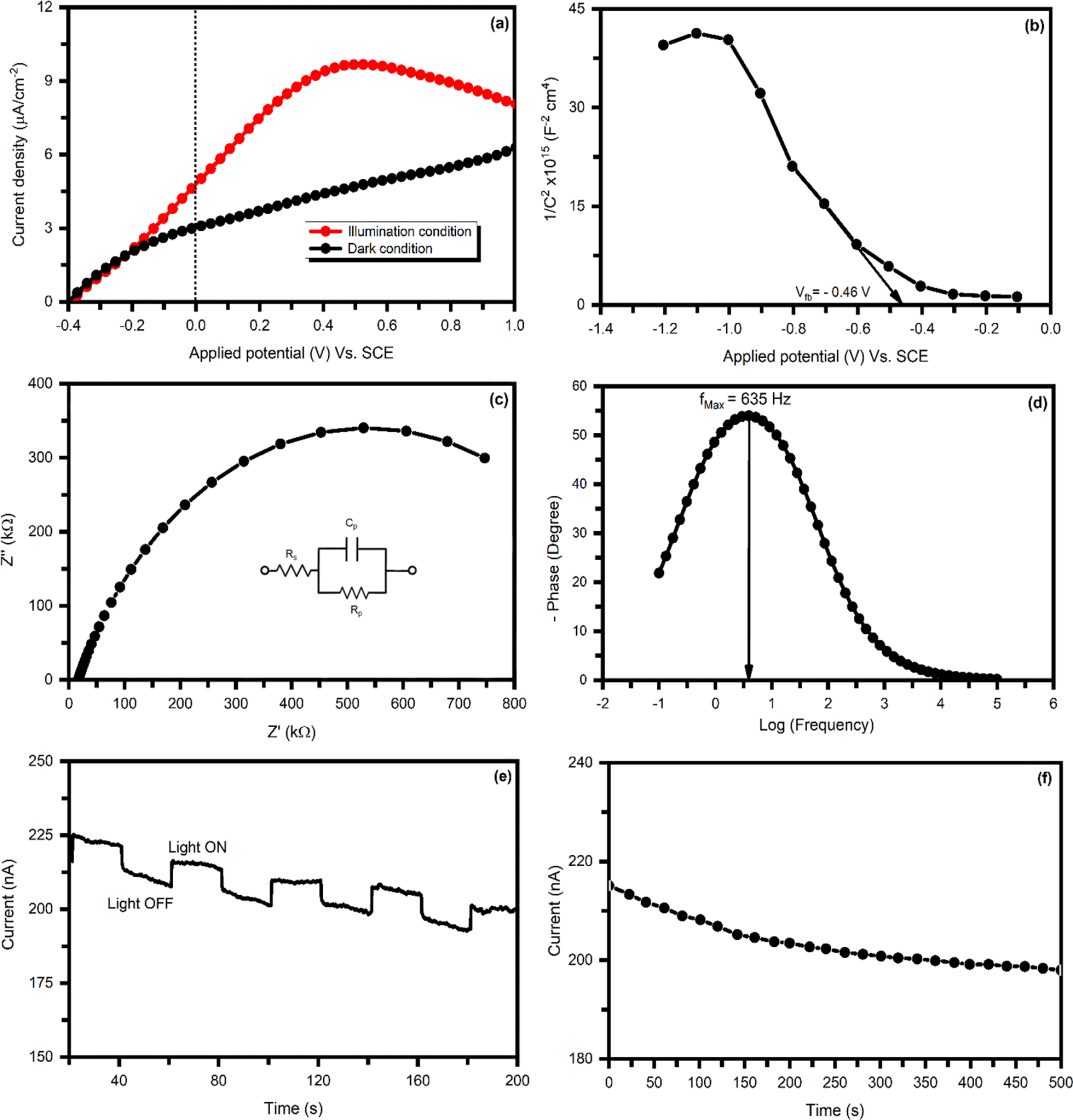
Photoelectrochemical (PEC) activity. (a) Variation of photocurrent density as a function of applied potential. (b) Mott–Schottky plot. (c) Nyquist plot. (d) Bode plot. (e) Chronoamperometric light switching behavior. (f) Stability curve of the ZrBi_2_Se_6_ nanoflower photoelectrode.

The ZrBi_2_Se_6_ nanoflower photoelectrode was systematically investigated using LSV for its PEC activity. The measurements were conducted with a three-electrode system at the potential window between −0.4 and 1.0 V vs SCE in a 0.5 M Na_2_SO_4_ (pH = 7) electrolyte. The graph of the photocurrent density (*J*) vs applied potential (*V*) is shown in Figure 9a. Due to a nonfaradaic reaction, a dark current was observed for the ZrBi_2_Se_6_ nanoflower photoanode while measuring the current density. However, under illumination, the photocurrent density increases with increasing applied potential. The highest photocurrent density of 9.7 μA/cm^2^ was observed for the ZrBi_2_Se_6_ nanoflower photoanode at 0.5 V of applied potential. It is worth noting that no photocurrent saturation was observed for a positive bias, indicating better charge separation upon illumination. The enhancement in photocurrent density can be attributed to the high surface area of ZrBi_2_Se_6_ nanoflowers in the vicinity of the electrolyte, which helps harvest many photons. This results in an increase in the charge carriers and, hence, in photocurrent density. The Mott–Schottky (MS) and EIS analyses further support this.

To study the semiconductor material for photoelectrochemical (PEC) water splitting application, the most important parameters are flat-band potential (*V*_fb_) and charge carrier density (*N*_d_). One of the simplest and most reliable methods for estimating *V*_fb_ and *N*_d_ values is the Mott–Schottky analysis. According to this theory, the value of capacitance *C* at the electrode–electrolyte interface at different potentials (*V*) is given by^52^
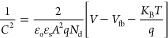
8where ε_o_ indicates the permittivity of free space, ε_s_ is the dielectric constant, *A* is an effective surface area of the semiconductor electrode, *q* is the electronic charge, *N*_d_ is the free charge carrier density, *K*_B_ represents Boltzmann’s constant, and *T* is the temperature in Kelvin. The flat band potential (*V*_fb_) and charge carrier density (*N*_d_) can be determined from the slope of the tangent of the MS plot. Using the values of *V*_fb_ and *N*_d_ in eqs 9 and 10, the depletion region width (*w*) can be determined.^53^
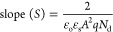
9
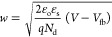
10

Figure 9b shows the MS curve of ZrBi_2_Se_6_ nanoflower photoanodes prepared using the solvothermal method. The estimated *V*_fb_, *N*_d_, and *w* values are −0.46 V, 4.8 × 10^15^ cm^–3^, and 1.85 nm, respectively. Furthermore, the high carrier density and electrical conductivity increase the PEC performance of ZrBi_2_Se_6_ nanoflower photoanodes. In addition, the significantly low depletion width (1.85 nm) of ZrBi_2_Se_6_ nanoflowers suggests simple diffusion of photogenerated charge carriers, which is promising for PEC activity.

The suitability of the charge transfer process across the ZrBi_2_Se_6_ nanoflower photoanodes and electrolyte interface was further revealed through EIS measurements. Figure 9c shows the Nyquist plots for ZrBi_2_Se_6_ nanoflower samples under visible light. The Randles equivalent electrical circuit was obtained from impedance data extracted by Nyquist plot as shown in the inset of Figure 9c. The fitted model includes the solution resistance *R*_s_ as a series and *R*_p_ inter-facial charge transfer resistance. *C*_p_ indicates the capacitance, which offers a constant phase element (CPE) as the non-ideal capacitor in the equivalent circuit model. *C*_p_ can result from the diffusion process related to the charge transfer reaction over the interface and the specific adsorption process of the different species on the electrode surface. In addition, the semicircle radius is correlated with the charge transfer capability occurring at the semiconductor electrode/electrolyte.^54^ The estimated charge transfer resistance values across the electrode–electrolyte interface and equivalent capacitance were found to be ∼730 kΩ and ∼40 nF, respectively. Therefore, for the suitability of a PEC application, the material must have low charge transfer resistance and a high equivalent capacitance value. However, a high equivalent capacitance value may also be responsible for the recombination of the charge carriers caused by the absorption of photon energy.

Variant impedance and phase-shift as a function of frequency can be revealed from the plot depicted in Figure 9d, which gives rise to the Bode plot. The Bode plot has prominent advantages over the Nyquist plot. The Bode plot indicates the complete transition from capacitive to resistive behavior. In the resistive behavior of the Randles-cell, the phase angle and impedance do not change with the frequency. In contrast, the imaginary component is minimal for low and high-frequency limits. Nevertheless, the cell behaves like having more capacitance for the intermediate frequencies, and impedance becomes frequency-dependent because the capacitor’s impedance begins to affect it. Therefore, due to significant imaginary components, the phase angle reaches 90°, and the cell is likely to behave as an inductor. The charge carrier (electron) lifetime (τ_e_) was calculated according to the equation^55,56^
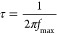
11

The calculated value of the lifetime of the charge carrier is found to be ∼0.25 ms.

Figure 9e indicates the time-dependent chronoamperometry photocurrent of ZrBi_2_Se_6_ nanoflowers measured under light ON and OFF at a time interval of 20 s. As seen, the photocurrent of ZrBi_2_Se_6_ nanoflower films abruptly changes under light ON and OFF conditions, showing effective photogenerated charge carrier separation. The nature of the time-dependent photocurrent curve is correlated with the light absorbing capacity and the ratio of surface to volume of ZrBi_2_Se_6_ nanoflowers. The estimated rise time (∼50 ms) and decay time (∼0.25 s) reveal the fast photocurrent response and excellent PEC performance of the ZrBi_2_Se_6_ nanoflower photoanode.

For applying ZrBi_2_Se_6_ nanoflowers as a photoanode, photoelectrochemical current stability is a critical parameter. Figure 9f shows the photocurrent versus time plot of the ZrBi_2_Se_6_ nanoflower photoanode recorded at 0.5 V of applied potential. It demonstrates the excellent stability of the ZrBi_2_Se_6_ nanoflower photoanode under the prevailing experimental conditions. These results further elucidate the potential candidature of ZrBi_2_Se_6_ nanoflowers as a photoanode for PEC activities.

Table 2 depicts the comparative analysis of ZrBi_2_Se_6_ nanoflowers as a photoanode with some binary material, heterojunction, and ternary material systems.

**Table 2:** Comparative Analysis of ZrBi_2_Se_6_ Nanoflowers as a Photoanode with some Binary Material, Heterojunction, and Ternary Material Systems

material and morphology	synthesis method	J (μA/cm^2^)	*V*_fb_ (V)	*N*_d_ (cm^–3^)	τ	*C*	reference
CdS nanospheres	hot Injection	12.0					(57)
CdSe nanocrystals	hot Injection	0.08	–0.12	1.30 × 10^16^	29.3 μs	34.5 nF	(58)
ZnS nanospheres	hot Injection	0.29	–0.39	6.54 × 10^16^	4.42 μs	0.12 nF	(59)
SnSe_2_ nanosheets	CVD	12.0	–0.42	2.25 × 10^20^	126 ms		(60)
ZnWO_4_ 1D rods	hydrothermal	32.0	+0.17				(61)
NiFe_2_O_4_ nanoplates	hydrothermal	42.0	+0.81				(61)
ZrBi_2_Se_6_ nanosheets	solvothermal	9.70	–0.46	4.80 × 10^15^	0.25 ms	40 nF	this work

Although the photocurrent density observed for the ZrBi_2_Se_6_ nanoflower photoanode is about 9.7 μA/cm^2^, the material still has a lot of scope for improvement and needs to be explored further. Nevertheless, after comparing, we found that the ZrBi_2_Se_6_ shows appreciable performance than some binary material, heterojunction, and ternary material systems. Thus, we believe that ZrBi_2_Se_6_ can be a promising material for photocatalytic water splitting applications.

**Figure 10:**
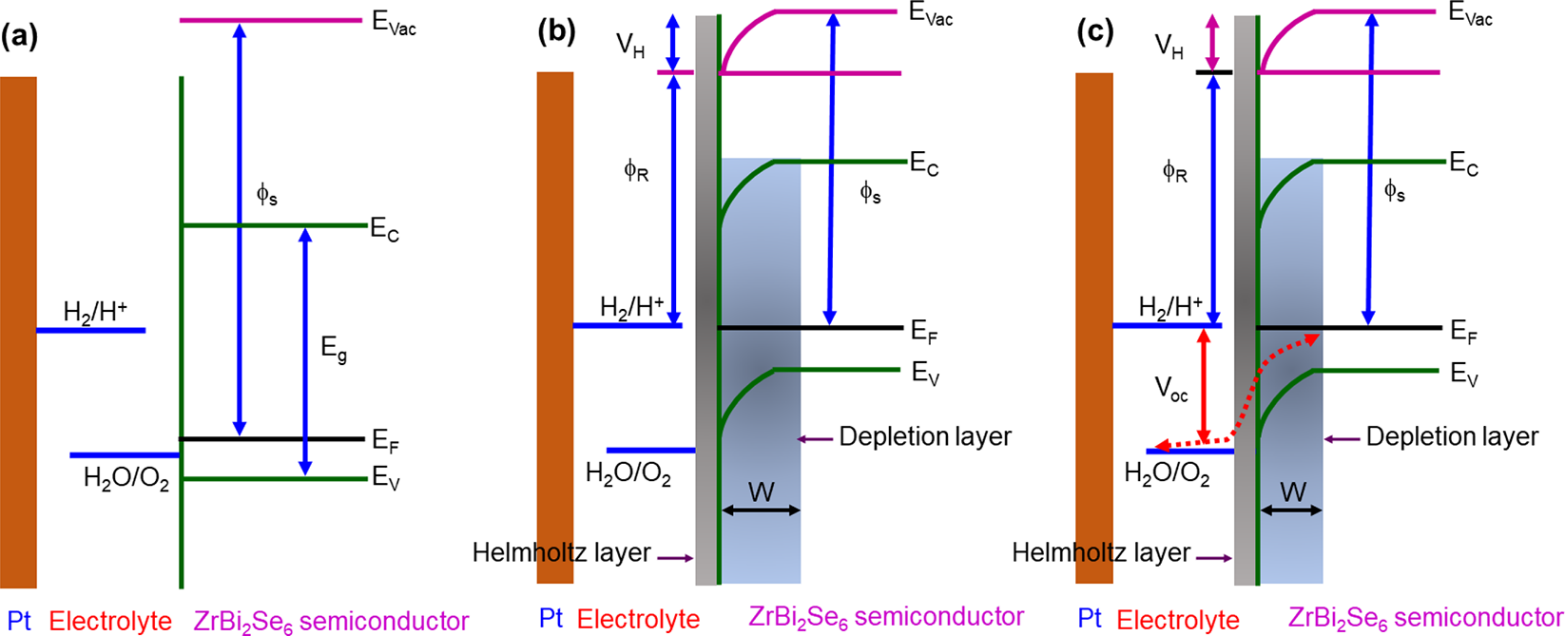
The band energy schematics of the p-type semiconductor (ZrBi_2_Se_6_ nanoflowers) and electrolyte (Na_2_SO_4_) interface indicates the relation between the electrolyte work function (ϕ_e_), electrolyte redox couple (H_2_O/O_2_ and H_2_/H^+^), semiconductor work function (ϕ_s_), Helmholtz layer potential drop (V_H_), and open circuit voltage (V_oc_) in three cases: (a) before equilibrium (b) after equilibration under dark conditions, and (c) in quasi-static equilibrium conditions under constant illumination.

Figure 10 represents the phenomenon of p-type ZrBi_2_Se_6_ nanoflower semiconductor/Na_2_SO_4_ electrolyte band-bending and the space charge region. Figure 10a–c illustrates the band energy schematic between the p-type ZrBi_2_Se_6_ nanoflower semiconductor/Na_2_SO_4_ electrolyte before, after, and quasi-static equilibrium conditions under constant illumination, respectively. When a p-type semiconductor photoanode is placed in an electrolyte, it contains a redox couple like H_2_O/O_2_, and an electron transfer occurs between the solution and the photoanode though equilibrium condition is obtained. After the equilibrium condition, the p-type semiconducting electrode has an extra positive charge, and the solution has an excess negative charge. These positive charges are distributed on the depletion layer with width (*w*), whereas the negative charges spread over a small region between the electrolyte and p-type semiconducting electrode known as the Helmholtz layer, as shown in Figure 10a,b. The steady-state illumination produces a nonequilibrium electron–hole pair, expressed by the quasi-Fermi energy level. The change in the quasi-Fermi level establishes an electric field near the p-type ZrBi_2_Se_6_ nanoflower semiconductor surface and gives rise to voltage and current. The photo-voltage (*V*_Ph_) or open circuit voltage (*V*_oc_) is the voltage produced by the built-in electric field of the semiconductor. Experimentally, it can be calculated by computing the potential difference between the hole and electron quasi-Fermi energy levels at no current flow.^62,63^ The maximum current generated in the built-in electric field is the short circuit current (*J*_sc_).

According to Jiang *et al.*,^63^ the water-splitting reaction requires a minimum Gibbs free energy of ∼237 kJ/mol. During the complete water splitting process, three major physiochemical processes occur. The first is the absorption of light by the semiconducting photoelectrode. A pair of charge carriers are created when a semiconductor (p- or n-type) absorbs photons. Therefore, the potential of the valence band for water oxidation should be more positive than that of the O_2_/H_2_O redox potential (1.23 V vs NHE, pH = 0), and the potential of the conduction band must be more negative than that of the H^+^/H_2_ redox potential (0 V vs NHE). In addition, the overpotential is also required to compensate for the energy losses related to the transportation of the photogenerated holes via the space charge region and electrons via the external circuit to the counter electrode. The second process is the efficient separation and transport of photogenerated electron–hole pairs with high mobility to avoid recombination of charge carriers in bulk or at the surface. The third and last process is the surface redox reaction for efficient water splitting. The potential of the charge carriers and the suitable kinetic reactions are necessary for efficient water splitting.
